# Preterm birth and stillbirth during COVID-19 pandemic in Bihor County/Romania

**DOI:** 10.3389/frph.2024.1286496

**Published:** 2024-02-29

**Authors:** Radu Galis, Paula Trif, Diana Mudura, Romina Murvai, Lucia Georgeta Daina, Florin Szasz, Rodica Negrini, Adrian Hatos, Beáta Fatime Gyarmati, Mandy C. Daly, Jan Mazela, Boris W. Kramer

**Affiliations:** ^1^ Department of Neonatology, Emergency County Hospital Bihor, Oradea, Romania; ^2^ Neonatology, Poznan University Medical Sciences, Poznan, Poland; ^3^ Doctoral School of Biomedical Sciences, University of Oradea, Oradea, Romania; ^4^ Department of Obstetrics and Gynaecology, Emergency County Hospital Bihor, Oradea, Romania; ^5^ Department of Psycho-Neuroscience and Recovery, Faculty of Medicine and Pharmacy, University of Oradea, Oradea, Romania; ^6^ Department of Obstetrics and Gynaecology, Faculty of Medicine and Pharmacy, University of Oradea, Oradea, Romania; ^7^ Doctoral School of Sociology, Faculty of Social Sciences, University of Oradea, Oradea, Romania; ^8^ Irish Neonatal Health Alliance, Wicklow, Ireland

**Keywords:** COVID-19 pandemic, lockdown, stillbirth, preterm birth, neonatal outcomes, Romania, epidemiological surveys

## Abstract

**Background:**

International studies have reported conflicting data about the effects of COVID-19 pandemic policy measures on maternal and neonatal health. A major impact was reported on stillbirth and prematurity. The published literature suggests that the economic setting influenced the effects of imposed mitigation measures with a more severe effect in low-income countries.

**Objectives:**

Our objective is to compare pregnancy outcomes at the only tertiary Maternity Hospital in Bihor County-Romania before and during the COVID-19 pandemic. This study aims to observe and document differences in perinatal outcomes across these periods, without inferring direct causation related to the pandemic or its associated restrictions.

**Materials and methods:**

We used data from the registries of Public Health Services Bihor to conduct a retrospective cohort analysis of preterm births and stillbirths during the COVID-19 pandemic in Bihor County, Romania. Pregnancy outcomes were compared between the pandemic period (March 2020–February 2022) to the corresponding historical pre-COVID-19 period (March 2018–February 2020). Maternal socio-demographic variables and neonatal characteristics of these periods were also examined.

**Results:**

The COVID-19 pandemic period was associated with an increase in the stillbirth rate (RR: 1.53, 95% CI, 1.05–2.23). Preterm birth was significantly impacted during this period and showed changes when analyzing gestational age (RR: 0.88, 95% CI, 0.79–0.96) or birth weight (RR: 0.91, 95% CI, 0.82–1.00). The main cause of stillbirth was intrauterine asphyxia due to placental causes (67.6%) or cord pathology (12.6%), the most frequently encountered maternal pathology was cardiovascular (28.3%) or infectious (21.7%). Our study revealed no significant changes in terms of maternal and neonatal characteristics during the two-year pandemic period.

**Conclusions:**

Lockdown restrictions in Bihor County, Romania were associated with an increase in stillbirths, whilst preterm birth rate decreased. This raises concerns about whether pandemic policy measures may have led to a failure in identifying and offering proper care for pregnant women who were more likely to experience an antepartum loss. Further studies across the globe are needed in order to integrate comparable data that will help develop adequate protocols and policies for protecting maternal and child health during the next pandemic that will follow.

## 1 Introduction

The COVID-19 pandemic and its associated lockdowns led to previously unimagined changes in modern daily life. The major burden was carried by health systems that underwent major restructuring to adapt to the additional workload generated by the COVID-19 crisis. Healthcare services prioritized emergency care, and in many places, all non-urgent admissions and surgeries were canceled during the strict lockdown periods (1). Pandemic-related restrictions, the effect on health services, together with a modified health service-seeking behavior may have also affected maternal health, perinatal care, and pregnancy outcomes. The research community took advantage of this unique natural experiment provided by the COVID-19 pandemic to identify new pathways for the reduction of preterm birth (PTB) and examine the impact of different factors previously incriminated in the etiology of prematurity (2–5). Speculation is widespread that the COVID-19 pandemic-related restrictions through the national lockdowns have indirectly impacted perinatal outcomes due to work and travel restrictions, fear of going to the hospital, and possible poor or delayed care. Previous studies reported inconsistent data regarding the effect of COVID-19 lockdowns on the PTB. During the early months of the pandemic, a reduction in PTB was reported in Denmark (4), the Netherlands (5), and Australia (6). Conversely, studies from Spain (7) or the USA (8) have not found such associations. In parallel, low- and middle-income countries (LMICs) reported an increase in stillbirth rates (9). However, only a few studies analyzed both PTB and stillbirth rates together as potentially competing perinatal outcomes. A first systematic review and meta-analysis (10) of 31 studies examining the impact of pandemic restrictions on perinatal outcomes confirmed a modest reduction in PTB in high-income countries (HIC) only, while the stillbirth rate was increased in the least developed countries. The analyses of data on PTB and stillbirth from 26 HIC and upper-middle-income countries (11) showed a subtle reduction of 3%–4% in the preterm birth rate among HIC but with no influence on the stillbirth rate. However, data from LMICs is still scarce, despite having the highest incidence of preterm birth and stillbirth.

In Romania, the first case of COVID-19 was detected on 26 February 2020 (12). As a result of additional cases being detected, a stringent nationwide state of emergency was declared in March 2020, with a mask requirement in all public spaces, a 2-m social distancing rule, prohibition of movement outside of household with very few exceptions, closure of schools and all non-essential public institutions, homework for private employers, and border closure to foreign visitors (13). In terms of health services, ambulatory care facilities closed and non-urgent admission and interventions were canceled to maximize the efficiency of healthcare personnel in COVID-19 cases. A gradual lifting of lockdown restrictions (reopening of schools, public institutions, as well as private businesses) began in May 2020, but a state of alert (strict functioning program for all restaurants, bars, and pubs; mass gathering limited to 50 persons for enclosed spaces; curfew during the night) remained in effect until March 2022 (14). Some of the mitigation measurements, e.g., school closure/reopening, varied across this period depending on the COVID-19 incidence rate of the county (14).

According to data published by the Romanian National Statistics Institute for 2021, there were 12.797 newborns with birth weight below 2,500 g born across the country, summing up 7% of all births. From the total number, only 5,352 (42%) resulted from pregnancy with a gestational age lower than 36 weeks, the majority being identified as term newborns with low birth weight (LBW) (15). Across European countries, LBW varies between 4.1% and 9.6%, with Romania being placed at the middle of this interval (16).

In 2021, Romania reported a stillbirth rate of 3.5‰ (639 cases), a rate that has suffered no significant variations since 2013. For 2019, WHO Europe reported a stillbirth rate of 4‰, while in Romania, it was as low as 3,2‰. We have therefore used the local data for comparison.

This study responds to the call of the International Stillbirth Alliance to report data on both PTB and stillbirths from LMICs, which have a higher burden of stillbirths (17). This is the first study intended to observe and document the impact of COVID-19-related restrictions on perinatal outcomes by providing local population-based estimates of perinatal care, PTB, and stillbirth rate during the pandemic compared with a historical period in a region of Romania.

## 2 Material and methods

### 2.1 Study setting

Healthcare in Romania is publicly funded, based on a social compulsory health insurance system. Access to healthcare is guaranteed by the Constitution of Romania. With the help of national health programs, different priority areas are covered, maternal and child health being included (12).

Maternity care is easily accessible, and all births are attended by midwives in collaboration with obstetricians and neonatologists or pediatricians.

Bihor County is Romania's 6th largest county, with a population of 600,000 people and a single tertiary maternity hospital.

### 2.2 Methods

The present study uses a retrospective cohort design to examine all preterm infants born and stillbirths in Bihor County between March 2018 and February 2022. We compared pregnancy outcomes (PTB and stillbirths) between two time periods: (1) 1st March 2018 and 29th February 2020 (before COVID-19) and (2) 1st March 2020 and 28th February 2022, the corresponding pandemic period, to avoid biases caused by seasonal variability. We also examined the relationships between maternal socio-demographic variables and neonatal characteristics of stillborn infants in Bihor County in the two periods. We proceeded to perform further analyses between two different periods: (1) 1st March–30th April 2020 (state of emergency in Romania) and 1st May 2020–28th February 2022 (state of alert in Romania) regarding stillbirths.

### 2.3 Data sources and study population

We identified and analyzed all births taking place between 1st March 2018 and 28th February 2022 in Bihor County. Preterm births and stillbirths occurring during this period were included in this study.

To ensure the relevance of the study population, the inclusion criteria consisted of all births within Bihor County during the specified time frame, with the eligibility criteria specifying preterm births meeting the gestational age or birth weight thresholds and stillbirths according to Romanian law.

Exclusion criteria comprised births outside the specified time frame and cases with missing or incomplete data on essential variables such as birth weight, gestational age, or stillbirth status.

Information on maternal socio-demographic variables and birth characteristics was obtained from public archives of the County Public Health Department and statistical reports of the Clinical County Hospital Bihor.

### 2.4 Definitions and measurements

The rate of preterm birth was defined as the number of infants born alive with a gestational age of 24^+0/7^ to 36^+6/7^ weeks, per 1,000 live births. The rate of stillbirth was defined as the number of infants born in the hospital with no signs of life and a gestational age of 28^+0/7^ weeks or more, per 1,000 births. This cutoff is according to Romanian law, where stillbirth is considered as fetal death at or after 28 weeks of pregnancy.

Gestational age was defined as the best estimate based on obstetric history, obstetric examination, and first prenatal ultrasound examination (18). Preterm infants were classified into moderate and late preterm infants (32^+0/7^–36^+6/7^ weeks gestation), very preterm infants (<32^+0/7^ weeks gestation), and extremely preterm birth (<28^+0/7^ weeks gestation). We also, classified preterm infants based on birth weight into moderate birth weight (<2,500 g), low birth weight (LBW; <2,000 g), very low birth weight (VLBW; <1,500 g), and extremely low birth weight (ELBW; <1,000 g).

Obstetric characteristics included maternal age, parity, type of labour (spontaneous or induced), mode of delivery, chorioamnionitis, and any other maternal pathology (e.g., maternal hypertension, maternal diabetes).

Social characteristics included educational level, employment, and residential area.

Indications to send the placenta for pathological examination in Bihor County include gestational age ≤36^+6/7^ weeks, intrauterine growth restriction, multiple births, congenital anomalies, fetal distress, abnormal fetal position, and stillbirths.

### 2.5 Statistical analysis

We computed Risk Ratio (RR) for all preterm births by gestational age and by birth weights, and for stillbirths.

To do this, we meticulously compared cases involving pregnant women exposed to the COVID-19 pandemic (1st March 2020–28th February 2022 period) with those unexposed, representing the period before the pandemic (1st March 2018–29th February 2020).

Our method involved initially determining the incidence of preterm births and stillbirths during the two defined periods: pre-COVID-19 (1st March 2018–29th February 2020) and during COVID-19 (1st March 2020–28th February 2022). Subsequently, we calculated the risk in each group by dividing the number of events (preterm births and stillbirths) by the total number of cases in the group (total births during the respective period in Bihor County). This was accomplished using the formula *Risk = number of events/total number of individuals in the group.* The final step entailed computing the risk ratio (RR) using the formula *Risk Ratio (RR) = risk in the exposed group/risk in the unexposed group.* By dividing the risk in the exposed group by the risk in the unexposed group, we obtained the risk ratio, offering a comprehensive understanding of the association between COVID-19 exposure and adverse pregnancy outcomes.

In our study, we handled deaths or live births occurring before 28 weeks by excluding them from the analysis of stillbirth cases. This decision was made to align with Romanian legislation, which defines stillbirth as fetal death at or after 28 weeks of pregnancy. However, it's important to note that births occurring before 28 weeks were included in the statistics related to preterm births but were not classified under the category of “stillbirth”. This approach ensures adherence to legal definitions while accurately representing the distinct categories of preterm births and stillbirths in our analysis.

In addition to the specific handling of deaths or live births occurring before 28 weeks in our study, we carefully considered other possible denominators based on the context of our research. Our primary denominators were the total number of births within Bihor County during the specified time frame, which allowed us to calculate rates and assess the impact of the COVID-19 pandemic period on adverse pregnancy outcomes. The inclusion of denominators such as total births in specific subperiods or subpopulations, allowed for a nuanced exploration of the associations and trends within our study.

We then performed *χ*^2^ or Fisher's tests for categorical variables and *T*-test for continuous variables to make comparisons and identify the relationships between our variables of interest in the case of stillbirths (*n* = 111).

The analysis was performed using IBM SPSS Statistics 27 software. The significance level for all the statistical tests was established at the *p*-value of .05.

### 2.6 Ethical approval

The internal review board of the County Public Health Department consented to the study and the publication of this data. The study was approved by the internal review board of the Clinical County Hospital Bihor.

## 3 Results

A total of 21.378 live births occurred in Bihor County between 1st March 2018–29th February 2022. From these, 11.305 were in the pre-COVID corresponding period (1st March 2018–29th February 2020) and 10.073 were during the pandemic (1st March 2020–28th February 2022).

In the period before the pandemic (1st March 2018–29th February 2020), there were 917 preterm births by gestational age, accounting for 8.11% of total births or 81.1 per 1.000 total births. During the COVID-19 period, this number decreased to 719, representing 7.13% of total births or 71.4 per 1.000 total births.

Similarly, for preterm births by birth weight, before the pandemic, there were 890 cases (7.87% of total births; 78.8 per 1.000 total births), while during COVID-19, there were 724 cases (7.18%; 71.8 per 1.000 total births).

**Figure 1:**
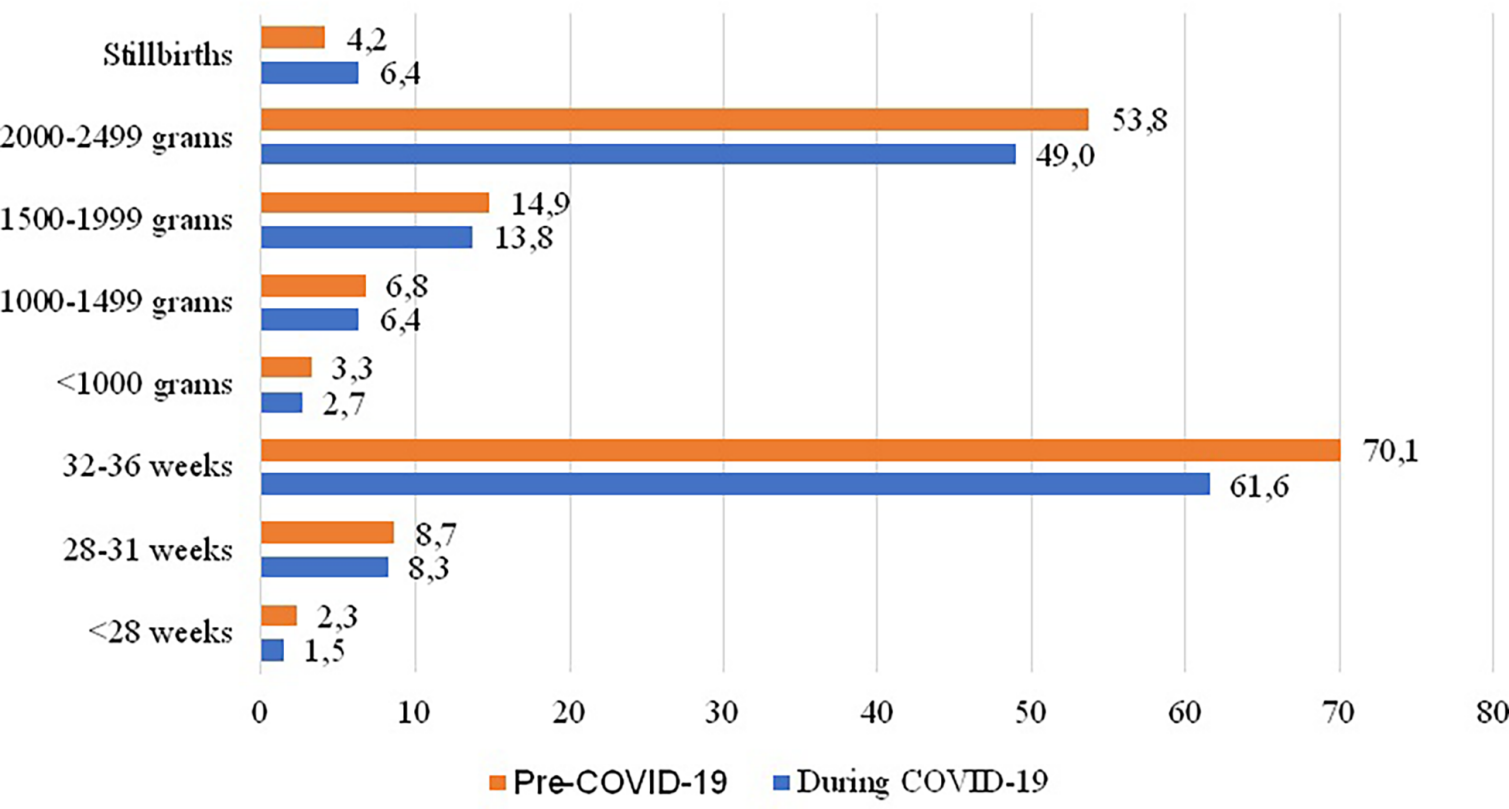
Rate of preterm births and stillbirth before and during the COVID-19 pandemic in Bihor County, 2018–2022 (No per 1,000 total births).

After calculating the Risk Ratio for preterm births, we found a risk ratio of 0.88 for preterm births by gestational age and 0.91 for preterm births by birth weight (Figure 1). This suggests that all preterm births were more likely to occur in the two years before the pandemic (1st March 2018–29th February 2020).

**Figure 2:**
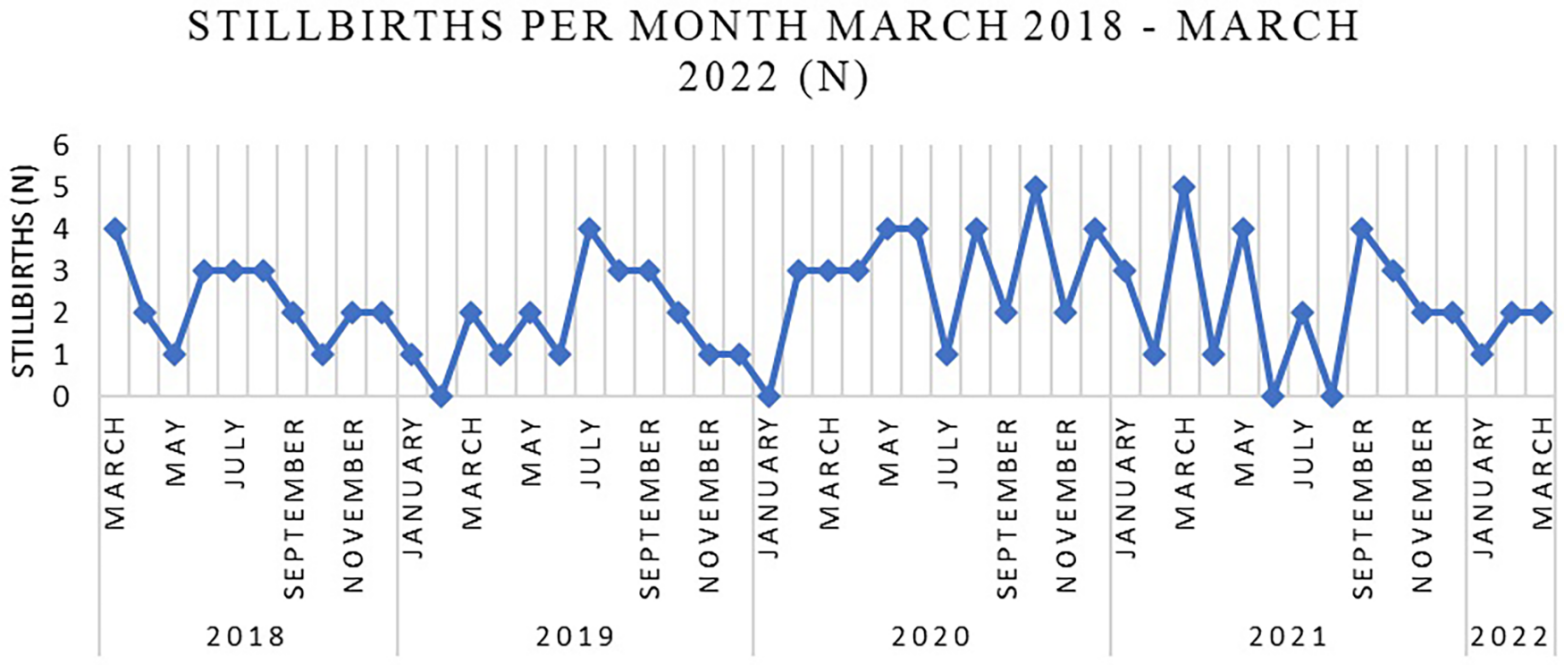
Stillbirths per month March 2018–February 2022 in Bihor County.

We also identified a greater risk that stillbirths occur 1.53 times more likely during the pandemic than in the corresponding years before (the risk during the pandemic is about 153% of the risk before de pandemic). Therefore, we analyzed the issue of stillbirths. Figure 2 shows the monthly variation in stillbirth cases from 1st March 2018 to 28th February 2022.

We then compared maternal and neonatal characteristics of stillborn infants between the two periods. Table 1 shows some of the maternal characteristics of stillborn infants like age, employment, educational level, and residential area.

**Table 1:** Maternal characteristics of stillborn infants before and during the COVID-19 pandemic period in Bihor County, 2018–2022.

	Before COVID-19 pandemic period	During COVID-19 pandemic period	*p*-value^a,b^
	March 2018–February 2020	March 2020–February 2022	
	(Stillbirth *n* = 47)	(Stillbirth *n* = 64)	
Maternal age (year) – *mean, SD*	29.57, 6.49	29.50, 7.72	.957^a^
Employment			.265^b^
− Student, *n* (%)	0 (0.0%)	2 (3.2%)	
− Employed, *n* (%)	15 (31.9%)	24 (38.1%)	
− Unemployed, *n* (%)	32 (68.1%)	35 (55.6%)	
− Unspecified, *n* (%)	0 (0.0%)	2 (3.2%)	
Educational level			.774^b^
− Primary school, *n* (%)	10 (21.3%)	14 (21.9%)	
− Secondary school, *n* (%)	11 (23.4%)	14 (21.9%)	
− High school, *n* (%)	8 (17.0%)	16 (25.0%)	
− Technical school, *n* (%)	2 (4.3%)	1 (1.6%)	
− Post-secondary education, *n* (%)	1 (2.1%)	2 (3.1%)	
− Higher education, *n* (%)	4 (8.5%)	8 (12.5%)	
− No education, *n* (%)	10 (21.3%)	7 (10.9%)	
− Unspecified, *n* (%)	1 (2.1%)	2 (3.1%)	
Residential area			.389^b^
− Rural, *n* (%)	32 (68.1%)	49 (76.6%)	
− Urban, *n* (%)	15 (31.9%)	15 (23.4%)	

^a^ Independent *t-*test.

^b^ *χ*^2^ test for independence.

The minimum age for mothers giving birth to stillborn infants was thirteen, while the maximum was forty-five. There was no significant difference regarding maternal age between the two periods [*t* (123.770) = .980, *p* = .329].

Regarding employment, we can see a greater difference between employed mothers (31.9% before the pandemic and 38.1% during the pandemic). Also, more unemployed mothers gave birth to stillbirth infants before the pandemic (68.1%) than during the pandemic (55.6%). However, these differences are not statistically significant.

The number of mothers who finished high school giving birth to a stillborn increased during the pandemic from 17.0% to 25.0%. The situation is similar for mothers with higher education (from 8.5% before the pandemic to 12.5% during the pandemic). The greatest difference regarding maternal level of education can be observed in the „no education” category – the percentage of mothers with no education giving birth to stillborn infants dropped from 21.3% to 10.9% during the pandemic. These differences are not statistically significant either.

There was a higher rate of mothers from the rural area during the pandemic (76.6% compared to 68.1%) and consequently a lower rate of those from the urban area (23.4% vs. 31.9%).

Table 2 shows the neonatal characteristics of stillborn infants from 1st March 2018 to 28th February 2022, focusing on the following variables: gestational age at birth, birth weight, sex, preterm birth, type of labor and birth, and the integrity of the membranes. We included in the analysis known risk factors for stillbirth, such as male gender of the infant or growth restrictions (low weight at birth).

**Table 2:** Neonatal characteristics of stillborn infants before and during the COVID-19 pandemic period in Bihor County, 2018–2022.

	Before COVID-19 pandemic period	During COVID-19 pandemic period	*p*-value^a,b^
	March 2018–February 2020	March 2020–February 2022	
	(Stillbirth *n* = 47)	(Stillbirth *n* = 64)	
Gestational age (week) *– mean, SD*	26.51, 12.41	27.73, 11.90	.601^a^
Birth weight (grams) – *mean, SD*	2,455.53, 877.56	2,170.47, 878.52	.094^a^
Low weight at birth (<2,500 grams), *n* (%)	23 (48.9%)	38 (59.4%)	.275^b^
Male sex, *n* (%)	22 (46.8%)	32 (50.0%)	.740^b^
Preterm birth			.891^b^
Extremely (<28 weeks gestation), *n* (%)	0 (0.0%)	1 (1.6%)	
Very (28–31 weeks gestation), *n* (%)	7 (14.9%)	8 (12.7%)	
Moderate (32–34 weeks gestation), *n* (%)	9 (19.1%)	12 (19.0%)	
Late (35–36 weeks gestation), *n* (%)	8 (17.0%)	10 (15.9%)	
Term birth (>37 weeks gestation), *n* (%)	8 (17.0%)	15 (23.8%)	
Unknown, *n* (%)	15 (31.9%)	17 (27.0%)	
Type of birth			.447^b^
− Vaginal birth, *n* (%)	23 (48.9%)	29 (45.3%)	
− Caesarean birth, *n* (%)	23 (48.9%)	35 (54.7%)	
Type of labor			.655^b^
− Pre-labor (latent phase), *n* (%)	4 (8.5%)	4 (6.3%)	
− Labor Onset, *n* (%)	4 (8.5%)	7 (11.1%)	
− Induced, *n* (%)	0 (0.0%)	2 (3.2%)	
− Spontaneous, *n* (%)	14 (29.8%)	19 (30.2%)	
− Expulsion, *n* (%)	1 (2.1%)	4 (6.3%)	
− No labor, *n* (%)	23 (48.9%)	24 (38.1%)	
− Another situation, *n* (%)	1 (2.1%)	3 (4.8%)	
Membranes			.085^b^
− Ruptured, *n* (%)	6 (13.0%)	13 (21.7%)	
− Intact, *n* (%)	40 (87.0%)	43 (71.7%)	
− Unspecified, *n* (%)	0 (0.0%)	4 (6.7%)	

^a^ Independent *t-*test.

^b^ *χ*^2^ test for independence.

The mean gestational age increased during the pandemic from 26.51 weeks to 27.73 weeks. Conversely, the mean birth weight dropped from 2,455.5 g before the pandemic to 2,170.4 g during the pandemic. These differences were found not to be statistically significant. 48.9% of the stillborn infants had low weight at birth before the pandemic, compared to 59.4% during the pandemic, which was also not significantly different.

More stillborn infants were born at term during the pandemic (23.8%) than before (17.0%). The number of cesarean sections increased during the pandemic from 48.9% (1st March 2018–29th February 2020) to 54.7%. Also, fewer women presented with ruptured membranes before the pandemic (13.0%) than during the pandemic (21.7%). Regardless, none of the mentioned differences were statistically significant (*p* > 0.05).

### 3.1 1st March 2020–28th February 2022

When referring strictly to the COVID-19 period in Romania (1st March 2020–28th February 2022), 10,073 live births occurred in Bihor County. Of these, 719 (7.13% from all live births; 71.4 per 1.000 live births) were preterm births by gestational age and 724 (7.18% from all live births; 71.8 per 1,000 live births) by birth weight. We had no precise information on the exact figures for each month.

Instead, we have conducted comprehensive analyses of stillbirths between the two periods: emergency state (1st March 2020–30th April 2020) vs. alert state (1st May 2020–28th February 2022). As mentioned before, we identified a significantly increased risk of stillbirths occurring during the COVID-19 pandemic than before – stillbirths were 1.53, 95% CI (1.05–2.23) times more likely to happen during the pandemic than the exact 2 years before it. As in the previous analysis, we further compared maternal and neonatal characteristics of stillborn infants between the two periods. Table 3 shows the maternal characteristics of stillborn infants corresponding to these periods.

**Table 3:** Maternal characteristics of stillborn infants during the emergency state and alert state in Romania, in Bihor County.

	Emergency state	Alert state	*p*-value^a,b^
	(March–April 2020)	(May 2020–February 2022)	
	(Stillbirth *n* = 6)	(Stillbirth *n* = 58)	
Maternal age (year) – *mean, SD*	23.17, 5.60	30.16, 7.65	.034*^a^
Employment			.872^b^
− Student, *n* (%)	0 (0.0%)	2 (3.5%)	
− Employed, *n* (%)	3 (50.0%)	21 (36.8%)	
− Unemployed, *n* (%)	3 (50.0%)	32 (56.1%)	
− Unspecified, *n* (%)	0 (0.0%)	2 (3.5%)	
Educational level			.493^b^
− Primary school, *n* (%)	1 (16.7%)	13 (22.4%)	
− Secondary school, *n* (%)	2 (33.3%)	12 (20.7%)	
− High school, *n* (%)	2 (33.3%)	14 (24.1%)	
− Technical school, *n* (%)	0 (0.0%)	1 (1.7%)	
− Post-secondary education, *n* (%)	1 (16.7%)	1 (1.7%)	
− Higher education, *n* (%)	0 (0.0%)	8 (13.8%)	
− No education, *n* (%)	0 (0.0%)	7 (12.1%)	
− Unspecified, *n* (%)	0 (0.0%)	2 (3.4%)	
Residential area			.432^b^
− Rural, *n* (%)	4 (66.7%)	45 (77.6%)	
− Urban, *n* (%)	2 (33.3%)	13 (22.4%)	

^a^ Independent *t-*test.

^b^ *χ*^2^ test for independence.

* *p* < .05.

We observed that the mean for the mother's age is older during the alert period (30.16, compared to 23.17 in the emergency period). This difference is statistically significant [*t* (62) = −2.171, *p* = .034].

Regarding employment, we can see a greater difference between employed mothers (50.0% in the emergency state and 36.8% during the alert state). However, this difference was not statistically significant.

Education-related, the number of mothers who finished only secondary school giving birth to a stillborn was higher during the emergency state (33.3%, while in the alert state, it was only 20.7%). The same decreasing trend was observed in the case of mothers who finished high school, from 33.3% during the emergency state to 24.1% during the alert state. In contrast, the number of mothers with higher education suffering a stillbirth increased during the alert state, from none in the emergency state to 13.8% (alert state). Either way, these results were not statistically significant.

There was a higher rate of mothers from the rural area during the alert state (77.6% compared to 66.7%) and consequently, a lower rate of those from the urban area (22.4% vs. 33.3%) who gave birth to stillborn infants.

Table 4 shows the neonatal characteristics of stillborn infants from 1st March 2020–28th February 2022.

**Table 4:** Neonatal characteristics of stillborn infants during the emergency state and alert state in Romania, in Bihor County.

	Emergency state (March–April 2020)	Alert state (May 2020–February 2022)	*p*-value^a,b^
	(Stillbirths *n* = 6)	(Stillbirths *n* = 58)	
Gestational age (week) *– mean, SD*	29.75, 10.85	27.52, 12.08	.667^a^
Birth weight (grams) – *mean, SD*	1,931.67, 999.81	2,195.17, 871.06	.489^a^
Low birth weight (<2,500 grams), *n* (%)	4 (66.7%)	34 (58.6%)	.531^b^
Male sex, *n* (%)	1 (16.7%)	31 (53.4%)	.098^b^
Preterm birth			.738^b^
Extremely (<28 weeks gestation), *n* (%)	0 (0.0%)	1 (1.8%)	
Very (28–31 weeks gestation), *n* (%)	2 (33.3%)	6 (10.5%)	
Moderate (32–34 weeks gestation), *n* (%)	1 (16.7%)	11 (19.3%)	
Late (35–36 weeks gestation), *n* (%)	1 (16.7%)	9 (15.8%)	
Term birth (>37 weeks gestation), *n* (%)	1 (16.7%)	14 (24.6%)	
Unknown, *n* (%)	1 (16.7%)	16 (28.1%)	
Type of birth			.569^b^
− Vaginal birth, *n* (%)	3 (50.0%)	26 (44.8%)	
− Caesarean birth, *n* (%)	3 (50.0%)	32 (55.2%)	
Type of labor			.935^b^
− Pre-labor (latent phase), *n* (%)	0 (0.0%)	4 (7.0%)	
− Labor Onset, *n* (%)	1 (16.7%)	6 (10.5%)	
− Induced, *n* (%)	0 (0.0%)	2 (3.5%)	
− Spontaneous, *n* (%)	2 (33.3%)	17 (29.8%)	
− Expulsion, *n* (%)	0 (0.0%)	4 (7.0%)	
− No labor, *n* (%)	3 (50.0%)	21 (36.8%)	
− Another situation, *n* (%)	0 (0.0%)	3 (5.3%)	
Membranes			.268^b^
− Ruptured, *n* (%)	0 (0.0%)	13 (24.1%)	
− Intact, *n* (%)	6 (100.0%)	37 (68.5%)	
− Unspecified, *n* (%)	0 (0.0%)	4 (7.4%)	

^a^ Independent *t*-test.

^b^ χ^2^ test for independence.

The mean gestational age slightly decreased during the alert state from 29.75 weeks to 27.52 weeks. Conversely, the mean birth weight increased from 1,931.7 g during the emergency state to 2,195.2 during the alert state. These differences were found not to be statistically significant. 66.7% of the stillborn infants had low weight at birth during the emergency state, compared to 58.6% during the alert state. There is a notable difference between male infants: only 16.7% of all stillbirths were boys during the emergency state, compared to 53.4% during the alert state.

More stillborn infants were born at term during the alert state (24.6%) than before (16.7%). Regarding the type of birth (cesarean or vaginal) we found no differences except for a slight increase in cesarean births during the alert state, from 50.0% to 55.2%. Another aspect worth mentioning is that during the emergency state, all mothers giving birth to stillborn infants had intact membranes, compared to just 68.5% during the alert state. Also, more women had no labor during the emergency state (50.0%) than during the alert state (36.8%). Regardless, none of the mentioned differences were statistically significant (*p* > 0.05).

**Figure 3:**
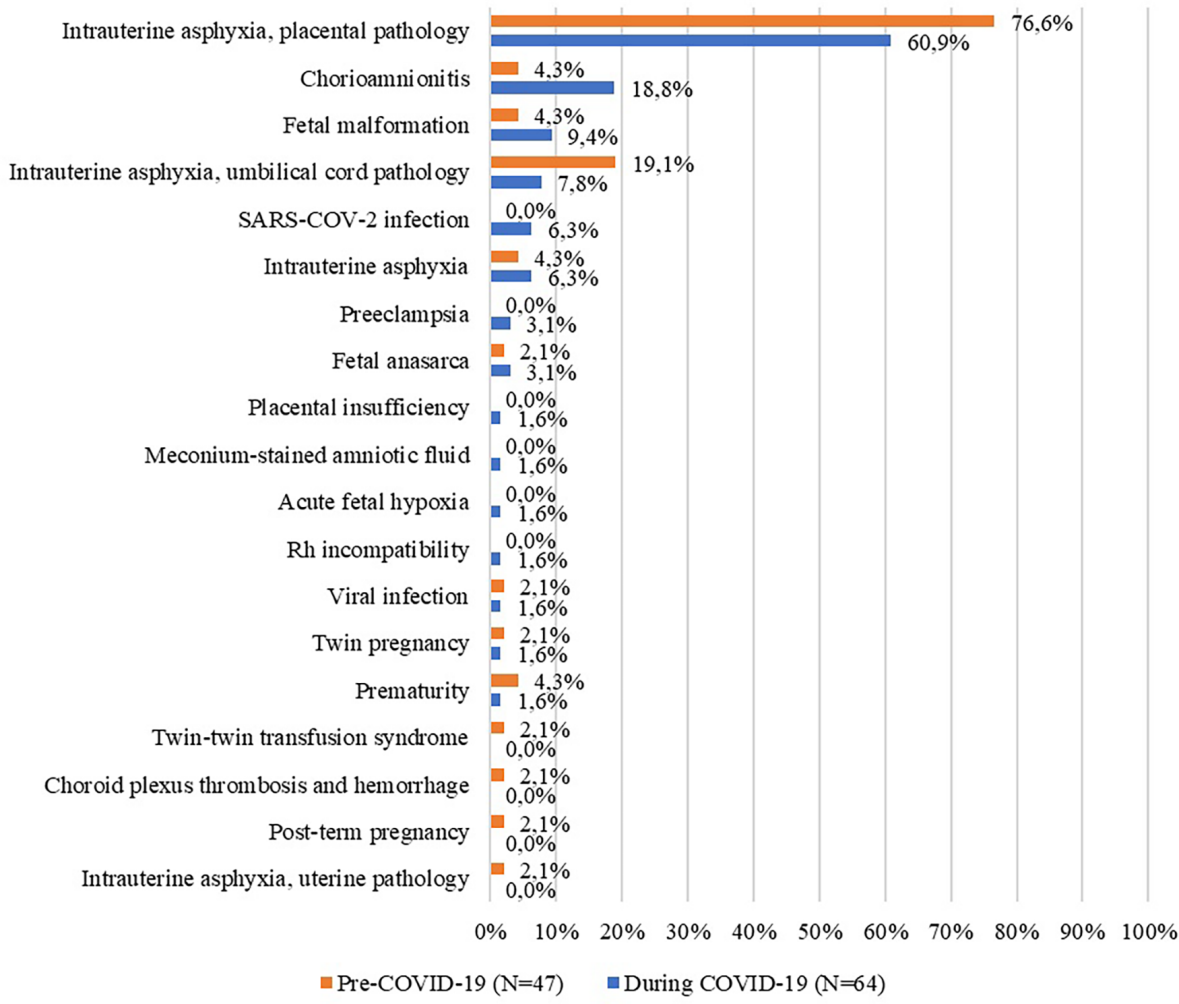
Diagnoses of the stillborn before and during the COVID-19 pandemic (%).

**Figure 4:**
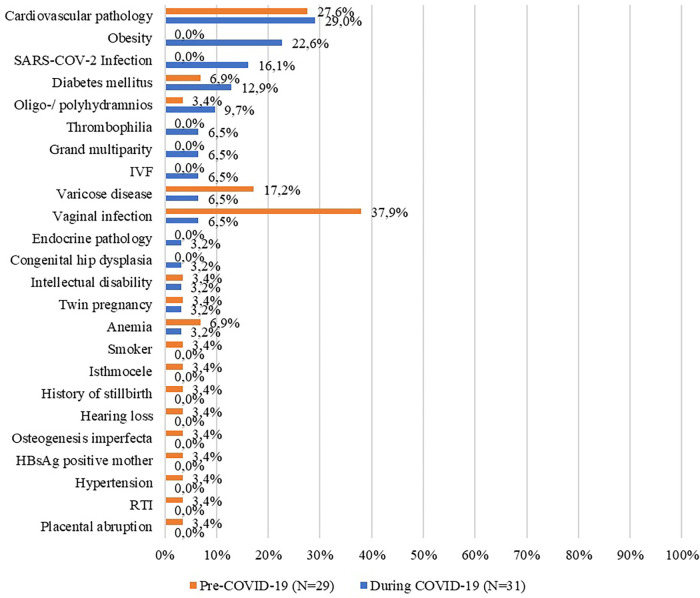
Maternal pathologies before and during the COVID-19 pandemic (%). IVF, *in vitro* fertilization; RTI, respiratory tract infections; HBsAg, hepatitis B surface antigen.

**Figure 5:**
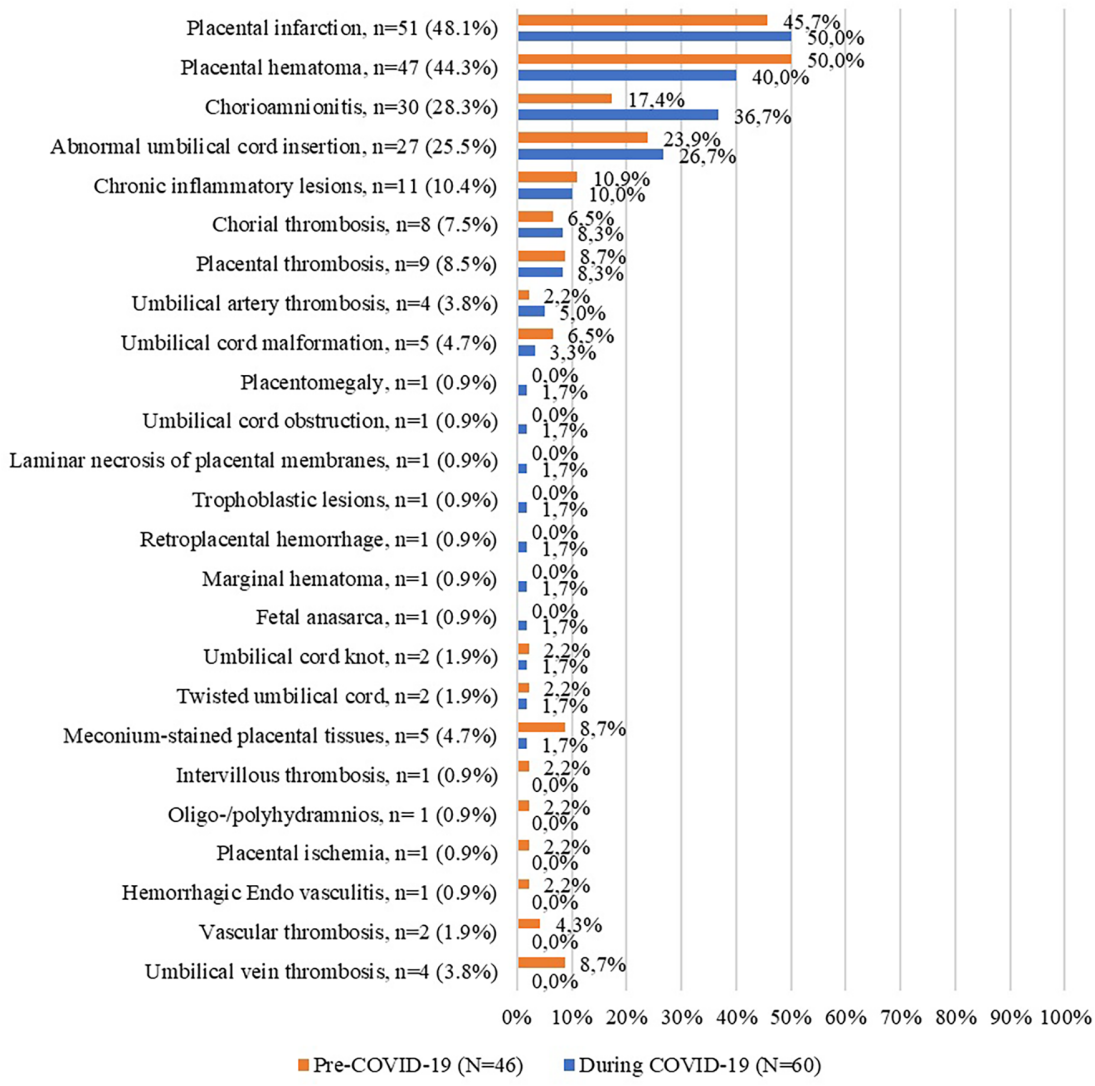
Pathological findings before and during the COVID-19 pandemic (%).

We also examined the diagnoses of the stillborn, pathological findings, and maternal pathologies. For the entire period (1st March 2018 –28th February 2022), we identified nineteen diagnostic entities, twenty-five different pathological findings, and twenty-three pathologies of the mother. The most frequent diagnostics for the entire period were the following: placental causes for intrauterine asphyxia (67.6%); cord pathology as a cause of asphyxia; and chorioamnionitis. In terms of the most frequently encountered pathological findings, we identified placental infarction (48.1%); placental hematoma and chorioamnionitis. The most common pathologies in mothers were cardiovascular disease; infections; varicose disease and obesity. The exhaustive list of the diagnosis of stillbirth, pathological findings, and maternal pathologies is presented in the figures below (Figures 3–5).

When we narrowed down the period to “during the pandemic” only, the figures changed and we were left with fifteen diagnostics, nineteen different pathological findings, and fifteen pathologies of the mother. Next, we analyzed these variables by period of emergency state and alert state. The most frequent diagnostics for the entire period were the following: placental pathology as a cause of intrauterine asphyxia (60.9%); chorioamnionitis (18.8%); malformed fetus (9.4%). In terms of the most frequently encountered pathological findings, they are identical to those for the entire period. The most common pathologies in mothers were cardiovascular disease; obesity; COVID-19 infection. In the following tables (Tables 5–7) the frequency of these diagnoses, pathological results, and maternal pathologies for both the emergency and the alert state imposed by the COVID-19 pandemic are presented.

**Table 5:** Diagnoses of stillborn infants during the emergency state and alert state, in Bihor County, 2018–2022.

Diagnoses of the stillborn	Pre-pandemic	Emergency state	Alert state
	(March 2018–February 2020), *n* (%)	(March–April 2020), *n* (%)	(May 2020–February 2022), *n* (%)
Intrauterine asphyxia, placental pathology	36 (76.6%)	3 (50.0%)	36 (62.1%)
Intrauterine asphyxia, umbilical cord pathology	9 (19.1%)	0 (0.0%)	5 (8.6%)
Chorioamnionitis	2 (4.3%)	1 (16.7%)	11 (19.0%)
Malformed fetus	2 (4.3%)	1 (16.7%)	5 (8.6%)
Intrauterine asphyxia	2 (4.3%)	1 (16.7%)	3 (5.2%)
SARS-CoV-2 infection	0 (0.0%)	0 (0.0%)	4 (6.9%)
Hydrops fetalis	1 (2.1%)	2 (33.3%)	0 (0.0%)
Prematurity	2 (4.3%)	0 (0.0%)	1 (1.7%)
Twin pregnancy	1 (2.1%)	0 (0.0%)	1 (1.7%)
Viral infection	1 (2.1%)	0 (0.0%)	1 (1.7%)
Preeclampsia	0 (0.0%)	0 (0.0%)	2 (3.4%)
Rh incompatibility	0 (0.0%)	0 (0.0%)	1 (1.7%)
Acute hypoxia	0 (0.0%)	0 (0.0%)	1 (1.7%)
Meconial cytotoxicity	0 (0.0%)	0 (0.0%)	1 (1.7%)
Placental insufficiency	0 (0.0%)	0 (0.0%)	1 (1.7%)
Intrauterine asphyxia, uterine pathology	1 (2.1%)	0 (0.0%)	0 (0.0%)
Post-term infant	1 (2.1%)	0 (0.0%)	0 (0.0%)
Choroid plex thrombosis and/or hemorrhage	1 (2.1%)	0 (0.0%)	0 (0.0%)
Twin-twin transfusion syndrome	1 (2.1%)	0 (0.0%)	0 (0.0%)

**Table 6:** Pathological causes related to stillbirth infants during the emergency state and alert state in Bihor County, 2018–2022.

Pathological results	Pre-pandemic	Emergency state	Alert state
	(March 2018–February 2020), *n* (%)	(March–April 2020), *n* (%)	(May 2020–February 2022), *n* (%)
Placental infarction	21 (45.7%)	4 (66.7%)	26 (48.1%)
Subchorionic hematoma	23 (50.0%)	2 (33.3%)	22 (40.7%)
Chorioamnionitis	8 (17.4%)	3 (50.0%)	19 (35.2%)
Abnormal umbilical cord insertion	11 (23.9%)	0 (0.0%)	16 (29.6%)
Inflammatory lesions	5 (10.9%)	2 (33.3%)	4 (7.4%)
Placental thrombosis	4 (8.7%)	0 (0.0%)	5 (9.3%)
Chorial thrombosis	3 (6.5%)	1 (16.7%)	4 (7.4%)
Meconium-stained placental tissues	4 (8.7%)	0 (0.0%)	1 (1.9%)
Malformed umbilical cord	3 (6.5%)	0 (0.0%)	2 (3.7%)
Umbilical artery thrombosis	1 (2.2%)	0 (0.0%)	3 (5.6%)
Twisted umbilical cord	1 (2.2%)	0 (0.0%)	1 (1.9%)
Umbilical cord knot	1 (2.2%)	0 (0.0%)	1 (1.9%)
Hydrops fetalis	0 (0.0%)	1 (16.7%)	0 (0.0%)
Marginal hematoma	0 (0.0%)	0 (0.0%)	1 (1.9%)
Retroplacental hemorrhage	0 (0.0%)	0 (0.0%)	1 (1.9%)
Trophoblastic lesion	0 (0.0%)	0 (0.0%)	1 (1.9%)
Focal necrosis	0 (0.0%)	0 (0.0%)	1 (1.9%)
Umbilical cord obstruction	0 (0.0%)	0 (0.0%)	1 (1.9%)
Placentomegaly	0 (0.0%)	0 (0.0%)	1 (1.9%)
Umbilical vein thrombosis	4 (8.7%)	0 (0.0%)	0 (0.0%)
Vascular thrombosis	2 (4.3%)	0 (0.0%)	0 (0.0%)
Hemoragic endovasculitis	1 (2.2%)	0 (0.0%)	0 (0.0%)
Intervillous thrombosis	0 (0.0%)	0 (0.0%)	0 (0.0%)
Placental ischemia	1 (2.2%)	0 (0.0%)	0 (0.0%)
Oligo polihidraminos	1 (2.2%)	0 (0.0%)	0 (0.0%)

**Table 7:** Maternal pathologies during the emergency state and alert state in Bihor County, 2018–2022.

Maternal pathologies	Pre-pandemic	Emergency state	Alert state
	(March 2018–February 2020), *n* (%)	(March–April 2020), *n* (%)	(May 2020–February 2022), *n* (%)
Cardiovascular pathology	8 (6.9%)	1 (25.0%)	8 (29.6%)
Infection	11 (37.9%)	1 (25.0%)	1 (3.7%)
Varicose disease	5 (17.2%)	1 (25.0%)	1 (3.7%)
Obesity	0 (0.0%)	0 (0.0%)	7 (25.9%)
Diabetes mellitus	2 (6.9%)	1 (25.0%)	3 (11.1%)
SARS-CoV-2 infection	0 (0.0%)	0 (0.0%)	5 (18.5%)
Oligo-/polyhydramnios	1 (3.4%)	0 (0.0%)	3 (11.1%)
Anemia	2 (6.9%)	0 (0.0%)	1 (3.7%)
IVF	0 (0.0%)	0 (0.0%)	2 (7.4%)
Twins	1 (3.4%)	0 (0.0%)	1 (3.7%)
Grand multiparity	0 (0.0%)	0 (0.0%)	2 (7.4%)
Intellectual disability	1 (3.4%)	0 (0.0%)	1 (3.7%)
Thrombophilia	0 (0.0%)	0 (0.0%)	2 (7.4%)
Congenital hip dysplasia	0 (0.0%)	0 (0.0%)	1 (3.7%)
Endocrine pathology	0 (0.0%)	0 (0.0%)	1 (3.7%)
Placental abruption	1 (3.4%)	0 (0.0%)	0 (0.0%)
Upper respiratory tract infection	1 (3.4%)	0 (0.0%)	0 (0.0%)
Smoking	1 (3.4%)	0 (0.0%)	0 (0.0%)
Anti-HBs positive mother	1 (3.4%)	0 (0.0%)	0 (0.0%)
Hypertension	1 (3.4%)	0 (0.0%)	0 (0.0%)
Osteogenesis imperfecta	1 (3.4%)	0 (0.0%)	0 (0.0%)
Hearing loss	1 (3.4%)	0 (0.0%)	0 (0.0%)
History of stillbirth	1 (3.4%)	0 (0.0%)	0 (0.0%)
Scar uterus	1 (3.4%)	0 (0.0%)	0 (0.0%)

IVF*, in vitro* fertilization.

## 4 Discussion

Our study assessed the perinatal outcomes during pandemics in a region in Romania demonstrating a significant increase in the stillbirth rate following COVID-19 mitigation measures. Although, the stillbirth rate for our County was 4.2‰ before the pandemic (2018–2020), close to the reported national stillbirth rate of 3.2‰ (19), in the following two years of COVID-19, this rate almost doubled, reaching 6.4‰. Data released by the National Public Health Institute show an increase in the stillbirth rate for the majority of the counties in our country for 2020 and 2021 (20).

Romanian national policy measures included mandatory testing of all symptomatic individuals and all persons requiring hospitalization during the entire pandemic period (21–28). Only 8.3% of the stillbirths reported in this study were among women COVID-19-positive. Although a direct consequence of SARS-CoV-2 infection at any time during pregnancy is possible, surveillance studies report that as much as 90% of COVID-19-positive pregnant women were asymptomatic (29). In this study, SARS-CoV-2 infection was a cause of death for only 3.6% of stillborn. Alternatively, the increase in stillbirths may have resulted from indirect effects such as reluctance of pregnant women to attend hospital when needed (e.g., with reduced fetal movements), fear of contracting infection, or reduced access to timely quality antenatal and intrapartum care (30–32). The risk profile for stillbirth during pandemics revealed by our study includes male sex, term or late preterm newborn with low birth weight. Birth weight below 2,500 g was associated with maternal malnutrition, especially in low- and middle-income countries, and increases the risk of infant mortality and morbidity, being universally accepted as an indicator of public health problems in a community (15, 33). Both prematurity and fetal growth restriction (FGR) lead to LBW and, if undetected antenatally, FGR increases stillbirth risk five times (34). We found an increase in LBW babies between the exposed and control groups; however, this was statistically not significant, suggesting that the rise in stillbirths was not because of an increase in the risk profile of our population.

Maternal characteristics of stillborn infants suffered no changes when referring to maternal age, educational level, employment, or residential area. The most prevalent maternal pathologies of stillborn were cardiovascular pathology, e.g., pregnancy-associated hypertension, preeclampsia, and infections, such as vaginal or amniotic fluid infection. For our County, chorioamnionitis almost doubled during 2020–2022 which warrants further research. An increased trend for malformed fetus during this period was also observed. However, pregnancy-associated pathologies may have been underdiagnosed during the pandemic because of changes in care-seeking behavior, but also due to institution-specific barriers to in-person care. This assumption was put forward in other studies coming from the Netherlands (5), Kenya (31) or UK (30). A meta-analysis done by Townsend et al. assessed the changes in maternity healthcare provision and healthcare-seeking by pregnant women during the pandemic and found that COVID-19 was a major factor that contributed to worsening pregnancy outcomes globally by hindering access to maternal healthcare services (32).

In terms of causes of stillbirth, intrauterine asphyxia due to either placental or cord pathology was the most frequent diagnosis, while anatomopathological data revealed infarction, hematoma, or chorioamnionitis-specific modifications of the placenta. Intrauterine asphyxia due to placental or cord pathology was also reported by the Romanian National Public Health Institute to be the leading cause of stillbirth countrywide in 2020 and 2021 (19, 20). Our findings are like those reported by other studies from low- and middle-income countries (Nepal, India, and Nigeria) and HIC (Australia, Italy) (9, 35–38).

Although pandemics are not new to humanity, the COVID-19 pandemic changed the world through the ampleness of the governmental measures taken to protect the population. During this unique experiment, the implication of various factors related to prematurity has been studied, offering the chance for a better understanding of the contribution of varied factors related to prematurity.

During the COVID-19 pandemic, many hospitals and healthcare facilities worldwide implemented restrictions and guidelines to minimize the spread of the virus and protect both patients and healthcare workers. NICUs had to solve an even more complex situation since the need for parental involvement, breastfeeding, and bonding conflicted with the safety of the infants and healthcare staff. The practice changes saw family-centered care, for which there is copious supporting evidence, obliterated or even ceased in many neonatal intensive care units (39, 40). Whilst preterm birth rate appeared unaffected by the pandemic according to some publications, healthcare practices changed enormously, and the long-term effects of this remain unquantified. Patient advocacies have raised awareness of this matter during the crisis and published prompt recommendations (41, 42).

In our region, the prematurity rate decreased during the 2 years pandemic period. Although our data does not allow for disaggregation of spontaneous and medically indicated preterm births, differences in the overall rate of prematurity were detected. In addition, the study outcomes may have competed for the end of pregnancy. Moreover, in our cohort, the risk ratio of being born prematurely during pandemics was 0.88, showing that preterm births were more likely to happen in the period before the pandemic. Our results differ from those of other studies reporting outcomes from Sweden (43) or Castilla-y-León (7), a Spanish region, that found no changes during the lockdown period, being similar to reports coming from Italy (44), the Netherlands (5), Iran (45) or a meta-analysis from China (46) that described a decreasing preterm birth rate. Data analysis of preterm birth in 26 HIC and upper-middle-income countries showed a reduction in prematurity rate only among HIC (10). The explanation for this variance across countries may lie in the differences in the COVID-19 mitigation measures, as well as the risk factors for prematurity from country to country.

## 5 Strengths and limitations

Our report has the strength of a detailed dataset presenting perinatal events together (PTB and stillbirths) up to the end of February 2022, when all mitigation strategies stopped in Romania. Moreover, for a better understanding of the COVID-19 implications on perinatal outcomes, we compared the pandemic cohort with a historically identical 2-year cohort, also reporting data regarding maternal characteristics and pathologies, diagnoses of stillbirths, and anatomopathological results for both periods (before COVID-19 pandemic period 2018–2020 and during COVID-19 pandemic period 2020–2022). The larger period considered allowed us in addition to study women who experienced changes in care and social activities for most or all of their pregnancy.

Limitations of this study include its retrospective nature, single-center setting, small numbers, and lack of data about antenatal care. It is also essential to address the potential impact of certain methodological choices on the estimation of rates here, specifically:

**Exclusion of stillbirths before 28 weeks** – the decision to exclude stillbirths occurring before 28 weeks from the analysis of stillbirth cases, aligning with Romanian legislation, may impact the overall estimation of stillbirth rates. This exclusion could potentially underestimate the true burden of adverse pregnancy outcomes, especially considering the critical gestational period defined by the legislation.

**Inclusion of births before 28 weeks in preterm births statistics –** while births occurring before 28 weeks were included in the statistics related to preterm births, this categorization may lead to potential overestimation of preterm birth rates. The choice to include these births in the preterm birth category, but not in stillbirths, may introduce a unique categorization bias that should be acknowledged.

**The absence of fetus-at-risk approach –** the decision not to employ the fetus-at-risk approach may impact the interpretation of our findings. This approach is known for providing a nuanced understanding of time-varying risks, and its absence in our study could limit the depth of our exploration into gestational age-related associations. We did not consider using this approach because our study design and available data did not allow for a comprehensive application of this method. The fetus-at-risk approach often requires detailed information on the time of onset of the exposure, precise gestational age determination, and continuous monitoring throughout the entire pregnancy, factors that may not have been sufficiently captured in our dataset. Given these limitations, we opted for a more pragmatic approach in our analyses, taking into account the available data and study design constraints.

**Lack of sensitivity analysis** – the absence of a sensitivity analysis limits our ability to assess the robustness of our results under different scenarios or assumptions. This limitation should be acknowledged, and it is crucial to recognize that uncertainties in our data and methodology could influence the precision and generalizability of our findings. We did not perform sensitivity analysis due to constraints in data availability and resource limitations. Conducting a sensitivity analysis typically involves varying key parameters or assumptions to assess the robustness of study findings. In our case, the unavailability of certain data and resource constraints prevented us from executing this additional analytical step. While sensitivity analysis is valuable for exploring the impact of uncertainties, our study was designed to optimize available resources and focus on the primary objectives of investigating the association between COVID-19 exposure and adverse pregnancy outcomes. We acknowledge the importance of sensitivity analysis in enhancing the depth of statistical interpretation and will consider its inclusion in future research endeavors with improved data accessibility and resources.

We acknowledge the limitation of a small sample size, particularly the occurrence of only 6 stillbirth cases in the initial period. The comparisons between the emergency state and the alert state were conducted with the intention of exploring potential trends and variations during different phases of the pandemic. However, we recognize that the small sample size may limit the generalizability of these findings.

Adjusting for confounders was not possible for this sample size. This can lead to biased, inaccurate results, which is another limitation of our study.

By transparently addressing these limitations in the study, we aim to provide a comprehensive understanding of the potential impacts on the estimation of rates and encourage future research to further refine and validate our findings.

## 6 Conclusion

Our study showed an increase in stillbirths in Bihor County, Romania, during the COVID-19 pandemic compared to the pre-coronavirus-19 period. The prematurity rate was lower in the mitigation pandemic in our county. The associated increase in stillbirth may be a competing outcome of pregnancies at risk. Unfortunately, we have no information on whether access to health services, maternal pathophysiology, and behavior, or social and environmental factors were involved. Our study was intended to identify associations which need to be studied in subsequent research.

As information accumulates on the effects of the COVID-19 pandemic lockdown across the world, more and more heterogenous perinatal outcomes across countries become evident. Independent of the effect of the COVID-19 disease, pandemic-related restrictions were associated with significant changes in pregnancy outcomes.

Now that the SARS-CoV-2 pandemic is over, there is a critical opportunity to learn from the past and to begin planning for the next pandemic, independently of the possible cause (coronaviruses, Influenza virus or others). This might be as well the opportunity to include the specific patient population in the development of protocols and policies. Therefore, it is essential to create protocols and regulations that safeguard pregnant women and acknowledge them as a vulnerable population subgroup in order to address subsequent global health challenges that will follow.

## Data Availability

The original contributions presented in the study are included in the article/Supplementary Material, further inquiries can be directed to the corresponding author.
